# A systematic review of the provision and efficacy of patient and carer information and support (PCIS) interventions for patients with dementia and their informal carers

**DOI:** 10.1007/s40520-019-01428-8

**Published:** 2019-12-05

**Authors:** L. Miles, B. M. S. McCausland, H. P. Patel, J. Amin, V. C. Osman-Hicks

**Affiliations:** 1 Faculty of Medicine (Clinical and Experimental Sciences), University of Southampton, Southampton, UK; 2 Department of Psychological Medicine, University Hospital Southampton NHS Foundation Trust, Southampton, UK; 3 Academic Geriatric Medicine, University of Southampton, University Hospital Southampton NHS Foundation Trust, Southampton, UK; 4 Medicine for Older People, University Hospital Southampton NHS Foundation Trust, Southampton, UK; 5 National Institute for Health Research, Southampton Biomedical Research Centre, University Hospital Southampton NHS Foundation Trust, Southampton, UK; 6 Memory Assessment and Research Centre, Southern Health NHS Foundation Trust, Southampton, UK

**Keywords:** Dementia, Care-givers, Information, Social support, Inpatient, Outpatient

## Abstract

**Background:**

The NHS dementia strategy identifies patient and carer information and support (PCIS) as a core component of gold-standard dementia care. This is the first systematic review of PCIS, performed to analyse the literature and evidence for these interventions.

**Aims:**

To systematically review literature evaluating the effectiveness of the provision of PCIS for people with dementia and their informal carers, in inpatient and outpatient settings.

**Methods:**

Searches of four online biomedical databases, accessed in September 2018. Studies were selected if they were: relating to people with dementia or their informal carers, based in inpatient or outpatient settings, published in English-language peer-reviewed journals no earlier than the year 2000 and assessed dementia-related information or social support interventions, by measuring qualitative or quantitative carer or patient-reported outcomes. Standardised data extraction and quality appraisal forms were used.

**Results:**

7 of 43 full-text papers analysed were eligible for analysis. 3 papers were different arms of one original study. Trends were present in the quantitative results towards reduced patient and carer depression and anxiety and the themes in the qualitative analysis were in favour of the intervention.

**Conclusions:**

The studies analysed were too heterogeneous in design, population and outcomes measured to make a conclusive opinion about the efficacy of these interventions. It is surprising that for such a common condition, a gold-standard evidence-based intervention and standardised delivery for provision of PCIS for people living with dementia in the UK does not exist. Further research is therefore vital.

**Electronic supplementary material:**

The online version of this article (10.1007/s40520-019-01428-8) contains supplementary material, which is available to authorized users.

## Introduction

Dementia is a significant and increasing public health problem, and a major cause of disability and dependency. Worldwide it affects around 50 million people, projected to rise to 152 million by 2050 [1]. A majority of people over the age of 60 rated Alzheimer’s as the disease they are most concerned about [2]. It has a significant impact on the UK National Health Service (NHS), with costs associated with dementia expected to more than double over the next 25 years [1, 3, 4], and 86% of patients over 75 admitted to hospital for over 72 h being identified as potentially having dementia [5].

Being a carer for an individual with dementia is associated with self-reported deterioration in physical and mental health. Perceived anxiety and depression in carers has been shown to be proportional to the severity of the care recipient’s dementia [4]. Many carers have limited understanding of dementia and the diseases that cause it [6]. This can result in stigmatisation and barriers to diagnosis and support [1]. Carer quality of life is reduced, perhaps as time for hobbies, social lives, romantic relationships and holidays progressively decreases, while sleep deprivation and burnout increase [6]. This is detrimental to both parties as the level of care decreases with increasing ill-health of carers [7].

It is widely recognised at both national and individual levels that patient and carer information and support (PCIS) are key in delivering gold-standard dementia care [8–11]. Support from professionals should be available for both the patient and carer to ask questions, plan for the future and gain information on available treatments [9]. There is a requirement for good quality, individually tailored information and support that is accessible to diverse populations of patients and carers at diagnosis and onwards. Many carers feel that exchanging practical advice and emotional support with people in a similar situation would be valuable to them [9].

There is a surprising paucity of literature evaluating PCIS in clinical practice. A review by Selwood et al. [12] of carer outcomes following psychological interventions found that despite there being little high quality evidence, interventions such as individual behavioural management therapy may improve carer mental health. Similarly, a review by Sorensen et al. [13] concluded that on average, caregiver interventions may be beneficial in reducing care-giving burden. Both of these reviews focused on carers and did not measure patient outcomes or other aspects of information or support.

We therefore performed a systematic review to evaluate:

1. The effectiveness of the provision of dementia-related PCIS to both inpatients and outpatients with dementia and/or their informal carers.
2. Whether PCIS interventions impact patient and/or carer-reported outcomes and experiences.

## Methods

### Search strategy and selection criteria

We aimed to locate all peer-reviewed, published studies meeting the pre-determined selection criteria: (1) involving men and/or women over the age of 16 with any form of dementia; (2) published in the English language; (3) based in an inpatient or outpatient setting; (4) comparing provision of patient or carer information or social support with standard care; (5) measuring qualitative or quantitative patient or carer-reported outcomes; (6) based in an NHS setting; (7) published in the year 2000 or after. Case studies were excluded. Selection was not restricted to studies using validated dementia diagnostic tools as there is presently no gold standard available.

Searches of databases were carried out from their inception to the beginning of September 2018. Preliminary searches were carried out on eight biomedical databases (Medline, Embase, Web of Science, CINAHL, Pubmed, Scopus, Psychinfo, Cochrane). After assessing the results, search strategies were redefined and repeated on Medline, Embase, Psychinfo and CINAHL. Citation tracking was carried out by manually screening the reference lists of included studies. PRISMA reporting guidelines were followed, supplementary Appendix A [14].

### Search terms

MESH subject heading terms were used for ‘dementia’, ‘caregivers’, ‘family’, ‘patients’, and ‘hospitals’. The NOT prefix was used to exclude ‘nursing home’, ‘residential care’, ‘community-dwelling’, ‘day-care’ and ‘delirium’. See supplementary Appendix B for the full search strategy.

### Data extraction and quality appraisal

**Fig. 1:**
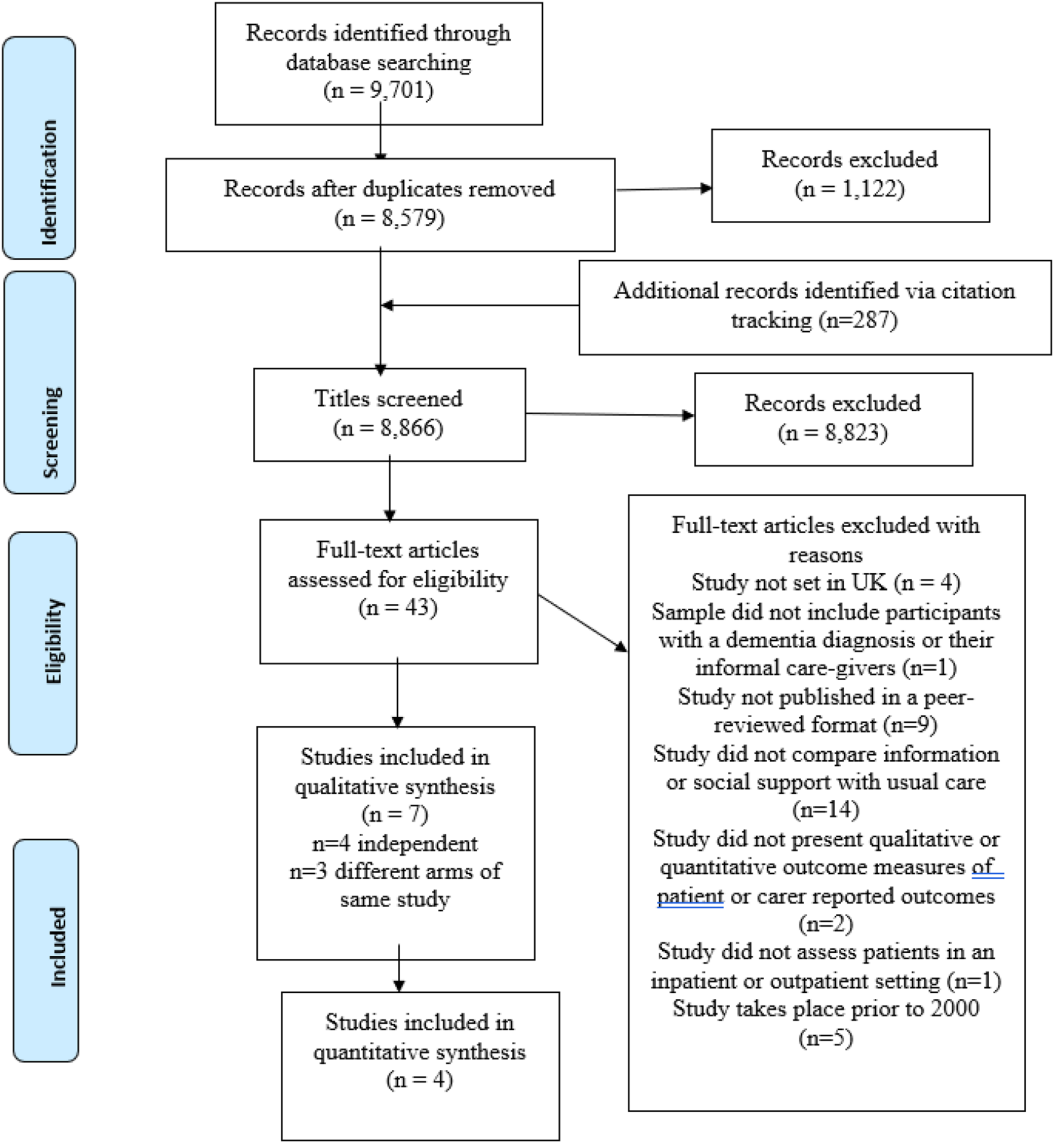
Flow diagram detailing search strategy, results of the selection criteria assessment, numbers of studies screened at excluded at each stage and reasons for exclusion of full-text articles

Endnote© online was used to collate the results of the searches that were screened against the inclusion and exclusion criteria. Full-text articles of potentially eligible studies were Iocated and assessed against the inclusion and exclusion checklists. A flow chart detailing the results of the searches and the reasons for exclusion is shown in Fig. 1. Data were extracted from eligible studies using a standardised data extraction form, Supplementary Appendix C. The quality of studies was assessed using a standardised appraisal form developed by Trevillion et al., using criteria adapted from validated tools [15–17], Supplementary Appendix D. A score out of 40 was given for each included study. Table 1 summarises all included studies and lists their appraisal scores.

**Table 1:** Summary of eligible studies

Trial	Trial design	Participant population	Intervention	Quality appraisal score
Cheston et al. [18]	Prospective cohort study	Males and females with diagnosis of any form of dementia	10 weekly hour-long sessions of The Dementia Voice Group Psychotherapy where participants were encouraged to share experiences with each other and reflect on their emotional significance	31
Cheston and Jones [19]	Parallel group study	Males and females with a diagnosis of probable Alzheimer’s disease according to the NINCDS-ADRDA criteria or probable vascular dementia according to the NINDS-AIREN criteria	10 weekly sessions of 75 min of psychotherapy or psychoeducation intervention	31
			Psychotherapy includes the discussion of experience and reflection upon emotional significance of these	
			Psychoeducation involves the discussion of set topics including power of attorney, relaxation, and memory strategies	
Marshall et al. [20]	Pilot randomised control trial	Males and females who have received a diagnosis of dementia from a consultant psychiatrist in a memory clinic	10 weekly sessions of a combination of psychotherapy and psychoeducation where participants are encouraged to share feelings and concerns	38
			They were given information about memory loss, dementia and medical treatments. The content of the sessions was paced to reduce distress	
Livingston et al. [21]	Randomised controlled trial	Male and female primary informal care-givers of family members referred to memory outpatient services	Eight sessions of psychoeducation. The topics included carer stress, dementia information, understanding and managing difficult behaviours, changing unhelpful thoughts, promoting acceptance, assertive communication, relaxation, planning for the future, increasing pleasant activities and maintaining the skills learnt	36
Livingston et al. [22]	Randomised controlled trial	Male and female primary informal care-givers of family members referred to memory outpatient services	24-month follow-up quantitative measurements following the intervention described in Livingston et al. [21]	36
Sommerlad et al. [24]	Qualitative study	Male and female primary informal care-givers of family members referred to memory outpatient services	24-month follow-up questionnaires about the intervention described in Livingston et al. [21]	29
Woods and Tadros (2013)	Service evaluation	Male and female carers of patients with dementia	Two weekly held sessions provided by Alzheimer’s Society support workers in the hospital where the study was based	16
			One session was an open access provision of information to anyone that attended. One was a drop-in session to meet carers and patients individually and discuss circumstances in more detail	
			These sessions consisted of provision of information, post-diagnosis counselling and on the spot emotional support	
			If required, participants could be referred to Alzheimer’s Society services and local support	

*NINCDS-ADRDA* National Institute of Neurological and Communicative Diseases and stroke—Alzheimer’s Disease and Related Disorders Association; *NINDS-AIREN* National Institute of Neurological Disorders and Stroke—Association Internationale pour la Recherché et l’Enseignement en Neurosciences

### Data analysis

The heterogeneity in outcomes measured by the included studies meant that quantitative data could not be pooled into a meta-analysis. We therefore used a narrative synthesis to present the data.

## Results

Seven studies were identified as eligible for analysis. The results are split by intervention used and whether patient or carer outcomes are presented. Five studies reported quantitative outcomes [18–22], the remaining study and service evaluation reported qualitative outcomes [23, 24]. The quantitative results are tabulated by patient or carer outcomes in Tables 2 and 3. An in-depth critical appraisal was conducted of the included studies and can be found in Appendix E.

**Table 2:** Patient-reported quantitative outcomes

Trial	Patient quality of life	Patient symptom severity	Patient self-esteem	Depression	Anxiety	Access to and ability to navigate services
Cheston et al. [18]	–	–	–	Significant decrease *p* = 0.034 (Cornell scale)	Borderline-significant decrease *p* = 0.05 (RAID scores)	–
				No significant change *p* = 0.241 (HADS depression)	No significant change *p* = 0.071 (HADS-anxiety)	
Cheston and Jones [19]	–	–	–	No significant change when adjusted for pre-intervention scores (Cornell scale, BASDEC)	No significant change when adjusted for pre-intervention scores (RAID, BAI)	–
Marshall et al. [20]	Non-significant evidence of improvement (QOL-AD)	Non-significant evidence of a reduction in cognitive functioning (MMSE)	Non-significant evidence of improvement (Rosenberg scale)	No change (Cornell scale)	–	Average contacts with NHS increased then decreased
						Use of social groups and day care increased
Livingston et al. [21]	Non-significant increase (QOL-AD)	–	–	–	–	–
Livingston et al. [22]	No change (QOL-AD)	–	–	–	–	–

*HADS* Hospital Anxiety and Depression Score, *RAID* rating of anxiety in dementia, *BASDEC* the Brief Assessment Schedule Depression Cards, *BAI* the Beck Anxiety Inventory, *QOL-AD* quality of life in Alzheimer’s disease, *MMSE* Mini-Mental State Examination. − denotes that the outcome was not measured by this study

**Table 3:** Carer-reported quantitative outcomes

Trial	Carer self-reported health status	Carer potentially abusive behaviour	Depression	Anxiety
Marshall et al. [20]	No significant changes (General Health Questionnaire)	–	–	–
Livingston et al. [21]	Significant improvement (4.09 mean score difference; 95% CI 0.34–7.83) (Health status questionnaire—mental health domain measures)	Non-significant decrease (Modified Conflict Tactics Scale)	Significant decrease (*p* = 0.02) (HADS)	
			Significant decrease in case-level depression (odds ratio (OR) 0.24; CI 0.07–0.76)	
			Case-level anxiety non-significant decrease	
Livingston et al. [22]	Significant improvement (7.47 mean score difference; 95% CI 2.87–12.08) (Health status questionnaire—mental health domain measures)	–	Significant decrease (*p* = 0.003) (HADS-T)	
			Significant decrease in case-level depression (OR 0.14; CI 0.04–0.53)	
			Case-level anxiety non-significant decrease	

*HADS* Hospital Anxiety and Depression Scale, *HADS-T* Hospital Anxiety and Depression Total Score. − denotes that the outcome was not measured by this study

### Psychological interventions

#### Patient-reported outcomes

Cheston et al. [18] ran 10-week psychotherapy groups for people with dementia and analysed outcomes at 6 weeks pre-intervention, at the start and end of the intervention and 10 weeks post-intervention. They found a significant decrease in depression scores using the Cornell scale (ANOVA analysis df = 3; *p* = 0.034; partial eta 0.147) but not when using the Hospital Anxiety and Depression Scale (HADS) (df = 3; *p* = 0.241; partial eta 0.074). For anxiety, there was a borderline-significant decrease in Rating Anxiety in Dementia (RAID) scores (df = 3; *p* = 0.050; partial eta 0.133) but not in HADS (df = 3; *p* = 0.071; partial eta 0.121).

Cheston and Jones [19] randomised people with dementia to psychotherapy or psychoeducation interventions. After adjustment for pre-intervention scores, there were no significant differences found in depression or anxiety scores using the Cornell or RAID scales, respectively; although the data for these adjusted scores were not presented in the original study.

A pilot study by Marshall et al. [20] informed a subsequent multi-centre trial, and therefore was not powered to identify significant results. They randomised patients to a 10-week group psychological intervention or a waiting-list control. Non-significant improvements were found in self-reported patient quality of life using the Quality of Life-Alzheimer’s Disease (QOL-AD) questionnaire (mean adjusted score difference 0.3; 95% confidence interval (CI) − 2.09 to 2.69), and in self-esteem using the Rosenberg self-esteem scale (mean adjusted score difference 1.58; CI − 0.08 to 3.25). Depression scores using the Cornell scale also showed a non-significant decrease (0.29 mean adjusted score difference; − 2.08 to 2.67). They noted that the intervention arm had a higher usage of social groups and day-centres, and that they used NHS services more frequently during the trial. This then decreased at the end of the study period which the authors feel may reflect longer-term NHS savings—by meeting more services early on and receiving appropriate support, thereby reducing the need for ongoing high-intensity service contacts.

Livingston et al. [21] conducted a randomised parallel group study with patients and their carers offering either a psychological coping strategy training intervention they titled START (StrAtegies for RelaTives) or normal care. They reported a non-significant increase in patient quality of life using the QOL-AD, as rated by the carer for the patient (mean score difference 0.59; CI − 0.72 to 1.89). At 24 months of follow-up, a non-significant difference was found in the QOL-AD scores for the patients (mean score difference 0.17; CI − 1.37 to 1.7) [22].

#### Carer-reported outcomes

Livingston et al. [21] found a significant decrease in carer HADS scores after the START intervention (− 1.8 adjusted point difference in means; CI − 3.29 to − 0.31; *p* = 0.02). They looked at the rates of case-level symptoms of anxiety and depression and found that carers post START had significantly reduced case-level depression (odds ratio (OR) 0.24; CI 0.07–0.76). There was a non-significant reduction in case-level anxiety (OR 0.3; 0.08–1.05). They also found a non-significant decrease in carer, self-reported, potentially abusive behaviours towards the patient using the Modified Conflict Tactics Scale (OR 0.47; 95% CI 0.18–1.23) [21]. These findings persisted at 24 months follow-up [22]; the mean HADS scores were reduced by 2.58 (95% CI − 4.26 to − 0.9; *p* = 0.003), case-level depression rates were reduced (OR 0.14; CI 0.04–0.53) and case-level anxiety differences were non-significant (OR 0.57; CI 0.26 to − 1.24).

Marshall et al. [20] measured carer health using the General Health Questionnaire, and found no significant difference following their 10-week group psychological intervention (0.15 mean adjusted score difference; − 4.56 to 4.86). However, Livingston et al. [21] reported a significant increase in carer mental health using the Health Status Questionnaire mental health domain measures (4.09 difference in mean score; 95% CI 0.34–7.83), which persisted at 24 months follow-up (7.47 mean difference; 95% CI 2.87–12.08) [22].

The study by Sommerlad et al. [24] was the qualitative arm of the Livingston 2013 and 2014 studies [21, 22], which used self-completed questionnaires to evaluate carer’s experiences of START. Thematic analysis identified four themes. The first was important aspects of the therapy; relaxation techniques were most often reported as useful with 22/75 (29.3%) carers continuing to use them. 18/75 (24.0%) reported that understanding the condition made it easier to cope with difficult symptoms, prepare for the future and improve communication. 11/75 (14.7%) mentioned that they welcomed advice on coping with behaviour and communication. 17/75 (22.7%) carers valued the interaction with therapists and being able to share their concerns. 10/75 (13.3%) reported a prolonged impact; empowering them to seek help and apply the techniques independently, accept their situation and to share techniques with friends or relatives.

The second theme was continued use after the end of the therapy; 50/75 (66.7%) continued to use the intervention. Reasons for discontinued use included: being too busy or tired, have too little time, forgetting the sessions, the care recipient passing away, feeling they needed the therapist for guidance and feeling the techniques were not relevant to their situation.

The third theme was unhelpful aspects and areas for improvement. 11/75 (14.7%) carers suggested improvements, including: wanting more sessions or a more gradual end to the sessions, being too time consuming, wanting support from other carers and organisations, wanting involvement of other family members, finding it difficult to find a private place and wanting more information about prognosis. There was an element of personal preference; some carers felt the techniques did not fit with their approaches or personality, and some did not like the relaxation CD.

The final theme was appropriate time for delivery of the intervention: 61/75 (81.3%) carers felt it was the right time (directly after or at the time of diagnosis). 8 carers wanted it earlier, to prevent making major decisions without adequate knowledge. Six reported that they would have preferred the therapy to be later; it was noted that these carers tended to be caring for people with milder dementia with a shorter median time from diagnosis to intervention than those who wanted it delivered earlier.

### Support and information provision

Woods and Tadros [23] conducted interviews with six carers following their intervention. This comprised Alzheimer’s Society support worker drop-in sessions for carers at an NHS Hospital to give information and arrange community follow-up. The drop-in service was used by 196 individuals, and 18 onward referrals were made to Alzheimer’s Society services. Analyses were performed regarding reasons for attending sessions and what carers hoped to gain. Emergent themes included information seeking and looking for “someone [to be] there when you hit that brick wall”. All six carers were in the early stages of accessing support and reported receiving what they had hoped for. None of the carers had previously been to Alzheimer’s Society support groups or cafes, but two were aware of how to access them. One carer-reported feeling “a lot more supported” by regular contact with the Alzheimer’s Society.

All of the carers thought the service was useful, would recommend it to others and thought it would be helpful in other hospitals. Having the drop-in sessions at the General Hospital proved to be essential, as 5/6 carers said they would not have contacted the Alzheimer’s Society otherwise. The carers were happy with the information given and receiving connections for follow-up. One carer who had had a previous negative experience with Alzheimer’s Society now reported being in favour of this service.

## Discussion

### Approaches used

Six of the seven studies used some form of psychological intervention: psychotherapy, psychoeducation and/or training to develop coping strategies. The idea of psychological therapy and counselling in dementia is to provide patients and their carers with strategies and outlets to help manage both the symptoms of dementia and the emotional experience of living with the disease [18]. These psychological approaches are based in the theoretical recovery model of mental health, “redefining identity, challenging stigma and helping people with dementia to work with their family to take responsibility for living well with their illness” (p. 528) [20].

The Cheston et al. research group for both the 2003 and 2009 studies used group psychotherapy sessions to discuss the emotional impact of a diagnosis of dementia, with the facilitator guiding group reflection [18, 19, 25]. They advocate for a holistic, psychotherapeutic approach to dementia care, as opposed to seeing psychotherapy as a separate treatment [26].

Two studies used psychoeducation, following the Bender et al. [27] structure. In general, psychoeducation varies from psychotherapeutic techniques, by providing information rather than facilitated reflection, such as teaching coping strategies to manage the emotional and practical sequelae of dementia [28]. Cheston and Jones [19] focused on practical information about power of attorney, relaxation and memory strategies; Marshall et al. [20] included sessions on medical treatments and information about dementia. Marshall et al. [20] combined psychoeducation with psychotherapy, drawing on learning from their previous studies [18, 19].

The Livingston et al., research team developed a one-to-one psychoeducational manual (START) for teaching coping strategies to carers, which was delivered by psychologists [21, 22, 24]. The focus was on “discussion of behaviours or situations that carers found difficult… behavioural management techniques, skills to take better care of themselves (including changing unhelpful thoughts), relaxation, increasing and assertive communication, promoting acceptance, sources of emotional support, and positive reframing”, as well as providing information about care and legal planning (p. 4). This was adapted from a ‘Coping with Caregiving’ programme from the United States of America [29].

Woods and Tadros [23] was the only study to use information provision not delivered as part of a psychoeducational programme. This intervention was open to the general public, but particularly aimed at those with dementia. As with the psychoeducational interventions, patients and carers were encouraged to ask questions to learn more about understanding and managing dementia.

A secondary but important benefit having a group intervention [18–20], is the development of support networks of people with shared experiences. This can help to reduce isolation, fear and hopelessness for both patients and their carers [30]. This may not be true of the one-to-one approaches by Livingston et al. [21, 22, 24] and Woods and Tadros [23]; however, the latter study did involve linking service users to further services in the community, which may have a similar effect.

### Efficacy of PCIS

The significant results presented by the included studies were:

1. Group psychotherapy was shown to significantly reduce patient depression as measured using the Cornell depression scale [18].
2. The START manual significantly reduced carer anxiety and depression scores as measured using the HADS scale [21].
3. Carer mental health was significantly improved following START, measured using the Health Status Questionnaire mental health domains [21].
4. Case-level depression rates were reduced in carers following START [21].
5. Results 2, 3 and 4 persisted at 24 months following START [22].

The lack of other significant results may suggest that quantitative outcome measures are hard to evidence for this type of intervention. Psychological impacts and the benefit of support and education are nuanced and difficult to measure. Statistically, there is evidence that PCIS has a significant impact on patient and carer depression and possibly anxiety (but not to a case-level or using anxiety specific scales). The effect on case-level anxiety, quality of life, self-esteem and abusive behaviours are not proven. START has been shown to be cost-effective and has been recommended for use throughout the NHS [22]. The qualitative data supports the use of PCIS but suggests that there is not a one-size-fits-all approach which can be used, as every patient with dementia and their carers will have different needs, preferences and responses [23, 24].

## Strengths and limitations of this review

This was a systematic review using standardised data extraction and quality appraisal forms, increasing the robustness of the results and reducing researcher bias. The study follows PRISMA reporting guidelines [13], Appendix A, to make it transparent and reproducible. The mixture of quantitative and qualitative data allows a more accurate representation of the ideas and concerns of participants following each intervention. Search strategies were tested and redefined to maximise inclusion of the relevant literature.

Time constraints meant restrictions had to be made on which papers were included in the review, such as being English-language, set in the NHS and published after 2000, which could have introduced reporting bias. Publication bias may have been introduced by preferential publication of studies with significant, positive results. Due to the heterogeneous nature of the studies’ designs and outcomes measured, a meta-analysis was not possible. The lack of significant data means that definitive conclusions cannot be drawn about the effectiveness of PCIS in inpatient or outpatient settings.

## Conclusions and future recommendations

The quantitative data collated in this review suggests that PCIS interventions are associated with reduced patient and carer depression and anxiety, and the qualitative data in the narrative synthesis leans towards a positive effect in carer and patient outcomes. However, firm conclusions about the efficacy of PCIS interventions cannot yet be made. It is surprising that for such a common intervention for individuals with dementia, a gold-standard, evidence-based and standardised delivery for the provision of PCIS does not exist. It is clear from the qualitative analysis that patients and carers view information and social support in a positive light, whether or not they make a significant difference to clinical outcomes. Therefore, healthcare trusts should take this into account when designing dementia care pathways and strategies for use in inpatient and outpatient settings.

Further research is needed with larger sample sizes, using standard outcome measurements to be able to generate significant or non-significant results and enable comparisons and conclusions about efficacy.

## Summary

### What is known already

Dementia is a significant and increasing health problem with a growing impact on the NHS. Both living with dementia and caring for someone with the condition has a great impact on physical, social and emotional well-being. Patient and carer information and support (PCIS) is recognised as a key area in delivering dementia care.

### What this review adds

There is a surprising lack of evidence supporting PCIS, in part due to lack of rigorous research measuring patient and carer outcomes following these interventions.

### Future research recommendations

PCIS is a part of the gold standard of dementia care that we should be striving for. To enable national roll out of effective and useful services, there needs to be further investigation into which are the most efficacious interventions, methods of delivery and settings in which they are best suited.

## Electronic supplementary material

Below is the link to the electronic supplementary material.

Supplementary material 1 (DOCX 234 kb)
