# Supplementary material for: A systematic review of the provision and efficacy of patient and carer information and support (PCIS) interventions for patients with dementia and their informal carers
Source: Aging Clin Exp Res. 2019 Dec 5;32(12):2439–48. doi: 10.1007/s40520-019-01428-8 (PMC7680317; doi:10.1007/s40520-019-01428-8)
Supplement: Supplementary file 1 — Supplementary material 1 (DOCX 234 kb) [file 40520_2019_1428_MOESM1_ESM.docx]

**APPENDICES FOR:**

**A systematic review of the provision and efficacy of patient and carer information and support (PCIS) interventions for patients with dementia and their informal carers.**

Miles, L^1*^, McCausland BMS^1,2*^, Patel HP^3-6^, Amin J^1,7^, Osman-Hicks VC^1,2^.

^1^University of Southampton Faculty of Medicine (Clinical and Experimental Sciences), ^2^Department of Psychological Medicine, University Hospital Southampton, ^3^Academic Geriatric Medicine, University of Southampton, University Hospital Southampton NHS Foundation Trust, ^4^Medicine for Older People Southampton, Hampshire, UK, ^5^Medical Research Council Lifecourse Epidemiology Unit, University Hospital Southampton, ^6^National Institute for Health Research Southampton Biomedical Research Centre, University Hospital Southampton NHS Foundation Trust, ^7^Memory Assessment and Research Centre, Southern Health NHS Foundation Trust

*Joint first authors.

**Address for Correspondence:**

Dr Beth McCausland (ORCID: 0000-0001-9096-3289)

Email: [bmccausland@doctors.org.uk](mailto:v.osman-hicks@nhs.net)

**Appendix A – PRISMA checklist (page 6)**

| **Section/topic** | **#** | **Checklist item** | **Reported on page #** |
| --- | --- | --- | --- |
| **TITLE** | | |  |
| Title | 1 | Identify the report as a systematic review, meta-analysis, or both. | 1 |
| **ABSTRACT** | | |  |
| Structured summary | 2 | Provide a structured summary including, as applicable: background; objectives; data sources; study eligibility criteria, participants, and interventions; study appraisal and synthesis methods; results; limitations; conclusions and implications of key findings; systematic review registration number. | 2 |
| **INTRODUCTION** | | |  |
| Rationale | 3 | Describe the rationale for the review in the context of what is already known. | 4 |
| Objectives | 4 | Provide an explicit statement of questions being addressed with reference to participants, interventions, comparisons, outcomes, and study design (PICOS). | 5 |
| **METHODS** | | |  |
| Protocol and registration | 5 | Indicate if a review protocol exists, if and where it can be accessed (e.g., Web address), and, if available, provide registration information including registration number. | 6 |
| Eligibility criteria | 6 | Specify study characteristics (e.g., PICOS, length of follow-up) and report characteristics (e.g., years considered, language, publication status) used as criteria for eligibility, giving rationale. | 6,7 |
| Information sources | 7 | Describe all information sources (e.g., databases with dates of coverage, contact with study authors to identify additional studies) in the search and date last searched. | 6 |
| Search | 8 | Present full electronic search strategy for at least one database, including any limits used, such that it could be repeated. | 7 |
| Study selection | 9 | State the process for selecting studies (i.e., screening, eligibility, included in systematic review, and, if applicable, included in the meta-analysis). | 7 |
| Data collection process | 10 | Describe method of data extraction from reports (e.g., piloted forms, independently, in duplicate) and any processes for obtaining and confirming data from investigators. | 7 |
| Data items | 11 | List and define all variables for which data were sought (e.g., PICOS, funding sources) and any assumptions and simplifications made. | 7 |
| Risk of bias in individual studies | 12 | Describe methods used for assessing risk of bias of individual studies (including specification of whether this was done at the study or outcome level), and how this information is to be used in any data synthesis. | 7 |
| Summary measures | 13 | State the principal summary measures (e.g., risk ratio, difference in means). | 7 |
| Synthesis of results | 14 | Describe the methods of handling data and combining results of studies, if done, including measures of consistency (e.g., I^2^) for each meta-analysis. | 7 |

| **Section/topic** | | **#** | **Checklist item** | **Reported on page #** |
| --- | --- | --- | --- | --- |
| Risk of bias across studies | | 15 | Specify any assessment of risk of bias that may affect the cumulative evidence (e.g., publication bias, selective reporting within studies). | Appendix E, supplementary material |
| Additional analyses | | 16 | Describe methods of additional analyses (e.g., sensitivity or subgroup analyses, meta-regression), if done, indicating which were pre-specified. | 7 |
| **RESULTS** | | | |  |
| Study selection | | 17 | Give numbers of studies screened, assessed for eligibility, and included in the review, with reasons for exclusions at each stage, ideally with a flow diagram. | 7, 8, 24 |
| Study characteristics | | 18 | For each study, present characteristics for which data were extracted (e.g., study size, PICOS, follow-up period) and provide the citations. | 8-13, 25-7 |
| Risk of bias within studies | | 19 | Present data on risk of bias of each study and, if available, any outcome level assessment (see item 12). | 25-7 quality appraisal scores |
| Results of individual studies | | 20 | For all outcomes considered (benefits or harms), present, for each study: (a) simple summary data for each intervention group (b) effect estimates and confidence intervals, ideally with a forest plot. | 28-31 |
| Synthesis of results | | 21 | Present results of each meta-analysis done, including confidence intervals and measures of consistency. | n/a |
| Risk of bias across studies | | 22 | Present results of any assessment of risk of bias across studies (see Item 15). | Appendix E, supplementary material |
| Additional analysis | | 23 | Give results of additional analyses, if done (e.g., sensitivity or subgroup analyses, meta-regression [see Item 16]). | n/a |
| **DISCUSSION** | | | |  |
| Summary of evidence | 24 | | Summarize the main findings including the strength of evidence for each main outcome; consider their relevance to key groups (e.g., healthcare providers, users, and policy makers). | 13-16 |
| Limitations | 25 | | Discuss limitations at study and outcome level (e.g., risk of bias), and at review-level (e.g., incomplete retrieval of identified research, reporting bias). | 17 |
| Conclusions | 26 | | Provide a general interpretation of the results in the context of other evidence, and implications for future research. | 17-19 |
| **FUNDING** | | | |  |
| Funding | 27 | | Describe sources of funding for the systematic review and other support (e.g., supply of data); role of funders for the systematic review. | 20 |

**Appendix B – Search terms (page 7)**

**This example was used on the Medline database:**

1. dementia/ or alzheimer disease/ or exp dementia, vascular/ or exp frontotemporal lobar degeneration/ or lewy body disease/ or exp "diffuse cerebral sclerosis of schilder"/
2. dementia.mp. [mp=title, abstract, original title, name of substance word, subject heading word, floating sub-heading word, keyword heading word, protocol supplementary concept word, rare disease supplementary concept word, unique identifier, synonyms]
3. alzheimer*.mp.
4. frontotemporal lobar degeneration.mp.
5. lewy body disease.mp.
6. schilder.mp.
7. caregiver*.mp.
8. carer*.mp.
9. famil*.mp.
10. spouse.mp.
11. patient*.mp.
12. Caregivers/
13. Family/
14. Patients/
15. information.mp.
16. education.mp.
17. support.mp.
18. advice.mp.
19. counselling.mp.
20. counseling.mp.
21. 1 or 2 or 3 or 4 or 5 or 6

## Appendix C – Checklist and Data Extraction Form (page 7)

Author name

|  |
| --- |

Paper title:

|  |
| --- |

| **Inclusion criteria** | **If yes tick box** |
| --- | --- |
| The study is published in a peer-reviewed format; government report, theses, dissertations, research report or another peer-reviewed format |  |
| The study utilises an eligible study design; randomised controlled trial, non-randomised controlled study, parallel group study, before and after study, interrupted time series study, cohort study, case review, case control study, cross sectional study, qualitative interview, focus group interviews, service evaluation |  |
| Participants in the study sample are 16 years old or older |  |
| The study sample includes people with dementia or their informal care-givers |  |
| The study is set in an inpatient or outpatient setting |  |
| The study compares the provision of dementia related information or social support to usual care |  |
| The study presents qualitative or quantitative outcome measures |  |
| The study involves an NHS setting |  |
| The study was published in the year 2000 or later |  |

| **Exclusion criteria** | If yes tick box |
| --- | --- |
| The study is published in non-gray literature; book, conference paper, editorial or other non-peer reviewed format |  |
| The study utilises an ineligible study design: case study |  |
| Participants in the study are aged 15 or younger |  |
| The study sample does not include people with dementia or their informal care-givers |  |
| The study is not set in an inpatient or outpatient setting |  |
| The study does not compare the provision of dementia related information or social support to usual care |  |
| The study does not present qualitative or quantitative outcome measures |  |
| The study does not involve an NHS setting |  |
| The study was published before the year 2000 |  |

**Please enter the dates of data collection:**

| Year of start of data collection |  |
| --- | --- |
| Year of end of data collection |  |

**Please select the study design:**

| **Study Type** | **If yes tick box(es)** | **Please specify if required** |
| --- | --- | --- |
| Randomised controlled trial |  |  |
| Non-randomised controlled study |  |  |
| Parallel group studies |  |  |
| Before and after studies |  |  |
| Interrupted time series studies |  |  |
| Cohort study |  |  |
| Case review |  |  |
| Case control study |  |  |
| Cross sectional study |  |  |
| Qualitative interview |  |  |
| Focus group interviews |  |  |
| Other (please specify) |  |  |

**Please select the study sample type:**

| **Study Setting** | **If yes tick box(es)** | **Please specify is required** |
| --- | --- | --- |
| Emergency Department setting |  |  |
| Mental healthcare setting (inpatient liaison service as part of acute trust) |  |  |
| Acute hospital/medical services |  |  |
| Acute neurology services |  |  |
| Other acute healthcare setting (please specify): |  |  |
| Outpatient clinic |  |  |
| Other |  |  |

**Please select the sampling method used in the study:**

| **Sampling Method** | **If yes, tick box(es)** | **Specify if required** |
| --- | --- | --- |
| Random sampling |  |  |
| Systematic sampling |  |  |
| Stratified sampling |  |  |
| Convenience sampling |  |  |
| Matched sampling (please provide details) |  |  |
| Quota sampling |  |  |
| Other (please specify) |  |  |
| Not specified |  |  |

**Study Population**

**Please enter the country(s) in which the study was conducted:**

**Please enter the number of males and females in the study sample:**

| **Sex** | **Number** |
| --- | --- |
| Males |  |
| Females |  |
| Not specified |  |

**Please enter details of the age of the study sample:**

|  | **Age (years)** |
| --- | --- |
| Youngest |  |
| Oldest |  |
| Mean |  |
| Standard deviation |  |
| Not specified |  |

**Please enter the study’s inclusion criteria:**

**Please enter the study’s exclusion criteria:**

**Please enter information about response rate:**

| Number approached to participate |  |
| --- | --- |
| Number who agreed to participate |  |
| Not specified |  |

**Please enter any comments about response rate:**

**Dementia**

**Please enter the definition of dementia used in this study:**

**Please select the categories of dementia recorded for the sample (tick as many as apply and provide further detail if available):**

| **Category of Dementia (ICD 10) [DSM-5]** | **If yes tick box(es)** | **Provide further detail if available (e.g., specific diagnostic code)** | **Please indicate whether disorder was assessed with a diagnostic or screening instrument** |
| --- | --- | --- | --- |
| Major Neurocognitive disorder [possible 331.9; probable 294.1x] |  |  |  |
| Mild Neurocognitive Disorder [331.83] |  |  |  |
| Dementia in Alzheimer's disease (F00) [331.0] |  |  |  |
| Vascular Dementia (F01) [290.40] |  |  |  |
| Dementia in other diseases classified elsewhere (F02) |  |  |  |
| Dementia in Pick’s disease (F02.0) |  |  |  |
| Dementia in Creutzfeldt-Jakob disease (F02.1) |  |  |  |
| Dementia in Huntington’s disease (F02.2)[333.4] |  |  |  |
| Dementia in Parkinson’s disease (F02.3)[332.0] |  |  |  |
| Dementia in human immunodeficiency virus disease (F02.4)[042] |  |  |  |
| Dementia in other specified diseases classified elsewhere (F02.8) |  |  |  |
| Frontotemporal Lobar Degeneration [331.19] |  |  |  |
| Lewy Body Disease [331.82] |  |  |  |
| Traumatic Brain Injury [907.0] |  |  |  |
| Prion disease [046.79] |  |  |  |
| Unspecified dementia (F03) [799.59] |  |  |  |
| Other (please specify) |  |  |  |
| Not specified |  |  |  |

**If specified, please enter the dementia assessment measure used for this study:**

**Please select the criteria against which dementia is assessed:**

| **Criteria** | **If yes tick box(es)** | **Specify if required** |
| --- | --- | --- |
| ICD-10 (or earlier versions) |  |  |
| DSM-5 (or earlier versions) |  |  |
| Not specified |  |  |

**Interventions**

|  | **Tick all those that apply** |
| --- | --- |
| **Patient information** |  |
| **Patient social support** |  |
| **Carer information** |  |
| **Carer social support** |  |

**Describe the intervention**

**Outcomes**

**Please select whether outcome measures are qualitative or quantitative (tick as many as apply):**

| Qualitative |  |
| --- | --- |
| Quantitative |  |
| Not specified |  |

**Please state what measure of patient outcome was used (tick as many as apply):**

| Quality of Life |  |
| --- | --- |
| Symptom severity |  |
| Distress |  |
| Functional ability |  |
| Self-reported health status |  |
| Self efficacy |  |
| Time spent waiting |  |
| Access to and ability to navigate services |  |
| Involvement in decision making |  |
| Knowledge of care plan and pathways |  |
| Quality of communication |  |
| Support to manage LT condition |  |
| Would they recommend the service to family and friends |  |

**If specified, please enter any additional information about patient outcome measures provided by this study:**

**Please state what measure of carer outcome was used (tick as many as apply):**

| Quality of Life |  |
| --- | --- |
| Symptom severity |  |
| Distress |  |
| Functional ability |  |
| Self-reported health status |  |
| Self efficacy |  |
| Time spent waiting |  |
| Access to and ability to navigate services |  |
| Involvement in decision making |  |
| Knowledge of care plan and pathways |  |
| Quality of communication |  |
| Support to manage LT condition |  |
| Would they recommend the service to family and friends |  |

**If specified, please enter any additional information about carer outcome measures provided by this study:**

**Please enter any notes about these outcomes (e.g., are disaggregated figures available for analysis, were odds ratio adjusted?)**

**Please enter the following raw data:**

| Total number of patients with dementia included in the analysis receiving information/ social support |  |
| --- | --- |
| Total number of carers included in the analysis receiving information/ social support |  |
| Total number of patients with dementia included in the analysis not receiving information/ social support |  |
| Total number of carers included in the analysis not receiving information/ social support |  |

****Please repeat the outcomes section if you have further estimated for subgroups****

**Please enter any further comments not covered elsewhere:**

**Appendix D – Quality appraisal form (page 7)**

Please complete part 1 for all study designs and complete the relevant sections for part 2, specific to study design.

Score the answer to each question by ticking 0, 1 or 2:

0 – study does not meet criteria/answer question

1 – Study partially meets criteria/gives a partially satisfactory answer to the question

2 – Study fully meets criteria/gives a fully satisfactory answer to the question

A – Cheston et al 2003

B – Cheston et al 2009

C – Marshall et al 2015

D – Livingstion et al 2013, Livingston et al 2014

E – Woods and Tadros 2013

F – Sommerlad et al 2014

| Screening questions | | | | Score | | | |
| --- | --- | --- | --- | --- | --- | --- | --- |
|  | Question | Comments | | 0 | | 1 | 2 |
| 1 | Did the study ask a clearly focused question?  *– Is the hypothesis/aim/objective of the study clearly described?*  *-Is the study question focused in terms of the outcomes considered?* | A | |  | | 1 |  |
|  |  | B | |  | |  | 2 |
|  |  | C | |  | |  | 2 |
|  |  | D | |  | |  | 2 |
|  |  | E | |  | | 1 |  |
|  |  | F | |  | |  | 2 |
| 2 | Is the study design appropriate for the research question? | A | |  | |  | 2 |
|  |  | B | |  | | 1 |  |
|  |  | C | |  | |  | 2 |
|  |  | D | |  | |  | 2 |
|  |  | E | |  | |  | 2 |
|  |  | F | |  | |  | 2 |
| Continue only if score on each of questions 1 and 2 is one or more | | | | | | | |
| Detailed questions | | | | | | | |
| Measurement of risk of selection bias | | | | | | | |
| 3a | Is the sampling method appropriate for the research question?  *Consider:*  *-The sampling method used (i.e. random selection of subjects)*  *- If applicable, is there appropriate selection of controls?* | | A | |  | 1 |  |
|  |  |  | B | |  |  | 2 |
|  |  |  | C | |  |  | 2 |
|  |  |  | D | |  |  | 2 |
|  |  |  | E | |  | 1 |  |
|  |  | | F | |  |  | 2 |
| 3b | Are subjects appropriately defined?  *Consider:*  *- Inclusion/ exclusion criteria specified*  *- Inclusion/exclusion criteria appropriate* | | A | |  |  | 2 |
|  |  |  | B | |  |  | 2 |
|  |  |  | C | |  |  | 2 |
|  |  |  | D | |  |  | 2 |
|  |  |  | E | | 0 |  |  |
|  |  | | F | |  | 1 |  |
| 3c | Is the sample size appropriate?  *Consider:*  *- Is the sample size justified?*  *- Were a sufficient number of cases selected?*  *- If applicable, were a sufficient number of controls selected?* | | A | |  | 1 |  |
|  |  |  | B | |  | 1 |  |
|  |  |  | C | |  |  | 2 |
|  |  |  | D | |  |  | 2 |
|  |  |  | E | | 0 |  |  |
|  |  | | F | |  |  | 2 |
| 3d | Is the study sample representative of the population of interest?  *-Do the authors assess the representativeness of the study sample?* | | A | |  | 1 |  |
|  |  |  | B | |  | 1 |  |
|  |  |  | C | |  | 1 |  |
|  |  |  | D | |  |  | 2 |
|  |  |  | E | |  | 1 |  |
|  |  | | F | |  | 1 |  |
| 3e | Does the level of non-participation risk introduce bias?  *Consider:*  *-Are key demographic characteristics of non-participants reported and compared against participants?*  *-Does the study report on the impact of non-participation?*  *-If applicable, rates of attrition reported* | | A | |  |  | 2 |
|  |  |  | B | |  | 1 |  |
|  |  |  | C | |  | 1 |  |
|  |  |  | D | |  |  | 2 |
|  |  |  | E | | 0 |  |  |
|  |  | | F | |  |  | 2 |
| 4 | Is the study setting appropriate to the aims of the research? (e.g. setting, location, relevant dates) | | A | |  |  | 2 |
|  |  |  | B | |  |  | 2 |
|  |  |  | C | |  |  | 2 |
|  |  |  | D | |  |  | 2 |
|  |  |  | E | |  |  | 2 |
|  |  | | F | |  |  | 2 |
| 5 | Is the method of data collection appropriate for the aims of the research? | | A | |  |  | 2 |
|  |  |  | B | |  | 1 |  |
|  |  |  | C | |  |  | 2 |
|  |  |  | D | |  |  | 2 |
|  |  |  | E | |  |  | 2 |
|  |  | | F | |  |  | 2 |
| Measurement of risk of reporting bias | | | | | | | |
| 6 | Are suitable/standard criteria used for measurement of dementia?  *Consider:*  *-Criteria of dementia diagnosis was clearly defined*  *-Potential for bias of measurement*  *-If measures piloted*  *- Standardised/pre-validated measures (score 2 points)*  *- Researchers developed their own measure (score 1 point)*  *- No details of measurement were provided (score 0 point)* | | A | | 0 |  |  |
|  |  |  | B | |  |  | 2 |
|  |  |  | C | |  |  | 2 |
|  |  |  | D | | 0 |  |  |
|  |  |  | E | | 0 |  |  |
|  |  | | F | | 0 |  |  |
| 7 | Are known confounders accounted for by study design?  *- Was consideration of confounding factors accounted for in study design?* | | A | |  | 1 |  |
|  |  |  | B | |  | 1 |  |
|  |  |  | C | |  |  | 2 |
|  |  |  | D | |  |  | 2 |
|  |  |  | E | | 0 |  |  |
|  |  | | F | | 0 |  |  |
| 8 | Are known confounders accounted for in the analyses? | | A | |  |  | 2 |
|  |  |  | B | |  |  | 2 |
|  |  |  | C | |  |  | 2 |
|  |  |  | D | |  |  | 2 |
|  |  |  | E | | 0 |  |  |
|  |  | | F | |  |  | 2 |
| 9 | Are the statistical tests used to assess the main outcomes appropriate?  *-Was there adequate adjustment for confounding in the analyses?*  *- Do the analyses adjust for different lengths of follow-up (if applicable)?* | | A | |  |  | 2 |
|  |  |  | B | |  |  | 2 |
|  |  |  | C | |  |  | 2 |
|  |  |  | D | |  |  | 2 |
|  |  |  | E | | 0 |  |  |
|  |  | | F | |  |  | 2 |
| 10a | Are the estimates reported with confidence intervals and in detail by sub-group (if appropriate)?  *- Were the findings reported clearly?* | | A | |  | 1 |  |
|  |  |  | B | |  | 1 |  |
|  |  |  | C | |  |  | 2 |
|  |  |  | D | |  |  | 2 |
|  |  |  | E | | 0 |  |  |
|  |  | | F | |  |  | 2 |
| 10b | Are statistically non-significant results presented? | | A | |  |  | 2 |
|  |  |  | B | |  | 1 |  |
|  |  |  | C | |  |  | 2 |
|  |  |  | D | |  |  | 2 |
|  |  |  | E | | 0 |  |  |
|  |  |  | F | | 0 |  |  |
| 10c | Are data for relevant variables complete? | | A | |  |  | 2 |
|  |  |  | B | |  |  | 2 |
|  |  |  | C | |  |  | 2 |
|  |  |  | D | |  |  | 2 |
|  |  |  | E | |  | 1 |  |
|  |  | | F | |  |  | 2 |
| 11 | Was the conduct of the fieldwork appropriate to the study setting?  *-Was the allocation of the interviewer/interpreter sensitive to the background of the participant?*  *-Were fieldworkers trained and supported to work with people who have dementia?* | | A | |  |  | 2 |
|  |  |  | B | |  |  | 2 |
|  |  |  | C | |  |  | 2 |
|  |  |  | D | |  |  | 2 |
|  |  |  | E | |  |  | 2 |
|  |  | | F | |  | 1 |  |
| 12 | Were ethical considerations appropriately considered?  -*Did researchers obtain informed consent from all participants?*  *- Did researchers take adequate precautions to safeguard participant anonymity and confidentiality?*  *-Did fieldworkers offer information about dementia support and referral options to all participants?*  -*Were fieldworkers appropriately trained to deal with participant distress?* | | A | |  | 1 |  |
|  |  |  | B | |  | 1 |  |
|  |  |  | C | |  |  | 2 |
|  |  |  | D | |  | 1 |  |
|  |  |  | E | | 0 |  |  |
|  |  | | F | | 0 |  |  |
| 13 | Do the findings support the conclusions? | | A | |  |  | 2 |
|  |  |  | B | |  |  | 2 |
|  |  |  | C | |  |  | 2 |
|  |  |  | D | |  | 1 |  |
|  |  |  | E | |  |  | 2 |
|  |  | | F | |  |  | 2 |
| 14 | Are the strengths and weaknesses of the research discussed? | | A | |  |  | 2 |
|  |  |  | B | |  |  | 2 |
|  |  |  | C | |  |  | 2 |
|  |  |  | D | |  |  | 2 |
|  |  |  | E | |  |  | 2 |
|  |  | | F | |  |  | 2 |

Calculate total score (out of a possible total of 40):

**Appendix E - Critical appraisal (page 8)**

The two studies by Livingston et al [21,22] will be analysed as one as they are different arms of the same original study.

Attrition rates

The attrition rates of the studies featured in this review were high, potentially introducing bias. A total of 778 participants were recruited across all included studies; 512 of these participants were included in the final analysis giving an overall attrition rate of 34.2%. Sommerlad et al [24] reduced attrition bias by sending questionnaires to those who withdrew from original intervention as well those who completed all stages.

Quality appraisal using validated scores

We have taken an arbitrary score of 30/40 to represent higher quality studies using the appraisal checklist by Trevillion et al [15-17]. This checklist acts as a guide to appraise the studies in a reproducible and transparent way where higher scores reflect higher quality studies. Five studies scored above 30 [18-22]. However, the checklist was developed for quantitative methodology appraisal, so the lower scores obtained by the qualitative studies by Woods and Tadros [23] and Sommerlad et al [24] may not be representative of their quality. The individual scores are summarised in Figure 2 and given in detail in Appendix C.

Sample sizes

The sample sizes of five studies were small, potentially affecting the representativeness and generalisability of their results; Cheston et al [18] recruited 42 participants but only 19 completed all three stages, in another study by Cheston et al [19] 18 participants were recruited and data analysed for 16. The study by Marshall et al [20] recruited 60 participants with 52 completing all 3 stages, Woods and Tadros [23] analysed a sample size of 6 out of a possible 196 who utilised the intervention and Sommerlad et al [24] collected data from 75 participants out of a potential of 132. However, the study by Marshall et al [20] was a pilot, exceeding the calculated requisite of 24 participants, and the studies by Woods and Tadros [23] and Sommerlad et al [24] were qualitative studies so required fewer participants. In contrast the studies by Livingston et al [21,22] had a large sample sizes, recruiting 260, with 140 still taking part in the intervention at 24 months. This resulted in sufficient power to enable significant differences to be detected with 90% power at a 5% significance level.

External validity

The external validity of six studies was limited by the following: two studies by Cheston et al [18,19] and the study by Marshall et al [20] that only included people with a Mini Mental State Examination (MMSE) score of at least 18, excluding people with severe symptoms of dementia. Livingston et al [21,22] excluded carers living over 1.5 hours from the research base, meaning their results may only representative of a specific geographical area. Woods and Tadros [23] did not specify their inclusion or exclusion criteria. Sommerlad et al [24] collected data via questionnaires which cannot be certain to represent the participants true thoughts and feelings towards the intervention.

However, some efforts were made to improve sample representativeness: Cheston et al [18] recruited via referrals from a range of health care sources, Livingston et al [21,22] included carers who did not fluently speak English by providing an interpreter and Marshall et al [19] included people with varied dementia sub-diagnoses. Furthermore, Sommerlad et al [24] allowed the participants to review the transcripts summarizing their responses to check they accurately represented their ideas.

Internal validity

The study by Woods and Tadros [23] did not include details on how they controlled for confounding variables, potentially decreasing the internal validity of their study. Cheston et al [18] adjusted for baseline variables and medication use and Cheston et al [19] analysed and adjusted for differences in the different therapists’ behaviours. Marshall et al [20] adjusted the results for baseline measurements, as did Livingston et al [21,22] in addition to adjusting for carer and patient health and well-being and the differential effects of treatment over time. They used logistic regression to identify if missing outcome data was influenced by baseline characteristics.

Sommerlad et al [24] found a statistically significant lower age of respondents than non-respondents to be the only significant baseline difference between the groups and used measures to reduce interpreter bias; sending transcripts to participants with the opportunity to amend them if they felt they weren’t representative of their true thoughts. Potential bias might have been introduced in the study by Cheston et al as the first author ran the therapy sessions that were being analysed [19].

Standardised criteria for measurement

Standardisation of procedures increases the reliability and validity of a study. Woods and Tadros [23] did not include detail of any standardisation of procedures. Cheston et al [19] and Marshall et al [20] used standardised criteria for diagnosing dementia. Cheston et al [18] used validated referral criteria and standardised the timetable for therapy sessions. Both studies by Cheston et al [18] used validated and standardised outcome and baseline variable measures, such as the Cornell Scale for Depression in Dementia.

Marshall et al [20] used a standardised therapy session manual and standardised methods of measuring the outcome. Livingston et al [21,22] also used standardised measurements of outcome, a standardised manual for therapy sessions and a standard checklist to rate an example session for fidelity to the manual. Similarly, Sommerlad et al [24] used a standardised questionnaire and a standard thematic framework for analysing transcripts.

Selection bias

A number of studies had a risk of selection bias. Because Cheston et al [18] recruited via referrals from a range of sources, there were no controls over who made the referrals, or on what basis and how they made the diagnosis of dementia. Similarly, Marshall et al [20] used memory clinic staff to approach potentially suitable participants which introduced subjectivity. Sommerlad et al [24] invited all the remaining participants from Livingston et al [20,21] to take part in their qualitative study which only represents the views of those who did not withdraw from the study. Woods and Tadros [23] did not outline their selection criteria or the method in which participants were recruited. Marshall et al [20] and Livingston et al [21,22] used pre-determined selection criteria and computer-generated randomisation, which decreased the risk of selection bias. Cheston et al 2009 [18] allocated participants to each arm of the study on the basis of who joined the intervention group first but does not specify the basis of which they were led to join the group; this leads to ambiguity in whether bias was introduced.

Observer bias

Steps were taken by the studies to reduce observer bias. Cheston et al [19] used two blinded, independent researchers to collect the data. Marshall et al [20] also used an independent, blinded researcher to collect data and the participants were asked to not disclose their allocation group. However, they didn’t specify whether the researchers carried out randomisation procedures which could have introduced observer bias. Livingston et al [21,22] used an independent clinical trials unit to carry out the randomisation of participants and had blinded data collectors.

Sommerlad et al [24] used two independent researchers to collect data and used software to code the transcripts, but didn’t specify about blinding. In the study by Woods and Tadros [23] the interviewer was not known to the carers but was known to some members of staff involved in the intervention, meaning interviewers were not completely independent of the study. Cheston et al [17] used the first author to lead the intervention groups; a potential source of bias. Furthermore, clinicians of varying roles and experience were used as co-facilitators in each group creating a difference environment for each intervention group. None of the studies could feasibly blind the participants; potentially resulting in a placebo effect in the intervention groups.

Methods of analysis

Results analysis and presentation varied. Cheston et al [18] presented significant and non-significant data, p-values and all the data for relevant variables but did not present 95% confidence intervals, meaning the precision of the results could not be measured. Marshall et al [20] presented data for all relevant variables along with 95% confidence intervals; however, the data was not powered to show statistical significance. Livingston et al [21,22] presented data for all relevant variables, significant and non-significant, with p values and 95% confidence intervals.

Sommerlad et al [24] and Woods and Tadros [23] both presented qualitative data. In contrast to Sommerlad et al [24], Woods and Tadros [23] lacked detail in the themes identified and the participant variables. Cheston et al [19] and Marshall et al [20] both used SPSS to analyse their data while Livingston et al [21,22] used STATA. Cheston et al did not identify the tool used for statistical analysis [18].

Ethics, consent and confidentiality

Marshall et al [20] and Livingston et al [21,22] detailed that they gained informed consent from the participants. Cheston et al [18] also detailed gaining consent but did not use the phrase ‘informed consent’. They specified that participants were told that they did not have to take part in the research to access the intervention, and thus were not coerced into recruitment. Cheston et al [19], Sommerlad et al [24] as well as Woods and Tadros [23] did not include details of how they gained consent from participants.

Only Sommerlad et al [24] included details of how the participant anonymity and confidentiality was maintained; they anonymised all quotations and only included non-specific demographic information. In all the studies analysed, the facilitators of the therapy received training and had healthcare backgrounds which imply they had experience with people with dementia. Cheston et al [18] had experienced facilitators and clinicians to run the therapy sessions and Cheston et al [19] used an experienced clinical psychologist to supervise. Marshall et al [20] used facilitators that were trained and had worked in memory clinics for at least a year as a health care worker or clinician. Livingston et al [21,22] included trained therapy leaders and clinical psychologists to supervise. The intervention described in Woods and Tadros [23] was run by Alzheimer’s Society support workers.

Marshall et al [20] offered the intervention to the control group once the study had ended; making the study more ethically sound. With regard to ethical approval; Cheston et al [19] was approved by a local research ethics committee and followed NHS research governance guidelines, Marshall et al [20] was ethically approved, followed National Research Council guidelines for good clinical practice in clinical trials, was carried out in accordance with Mental Capacity Act 2005 and followed the principles of European clinical trials. Cheston et al [18], Livingston et al [21,22], Woods and Tadros [23] and Sommerlad et al [24] did not include details of ethical approval.
